# Cell type-specific analysis of transcriptome changes in the porcine endometrium on Day 12 of pregnancy

**DOI:** 10.1186/s12864-018-4855-y

**Published:** 2018-06-14

**Authors:** Shuqin Zeng, Jochen Bick, Susanne E. Ulbrich, Stefan Bauersachs

**Affiliations:** 1 ETH Zurich, Animal Physiology, Institute of Agricultural Sciences, Zurich, Switzerland; 2 Department for Farm Animals, University of Zurich, Genetics and Functional Genomics, Clinic of Reproductive Medicine, Zurich, Switzerland

**Keywords:** Pig, Pregnancy, Estrous cycle, Uterus, Endometrium, Transcriptomics, RNA-Seq

## Abstract

**Background:**

Along with trophoblast elongation (Days 10 to 12), estradiol is secreted in increasing amounts for recognition of pregnancy. Endometrial secretions driven by ovarian progesterone and conceptus signals are essential for conceptus growth and development. Results of transcriptome analyses of whole endometrial tissue samples in the pig indicated the need for cell type-specific endometrial gene expression analysis for a better understanding of transcriptome changes associated with establishment of pregnancy.

**Results:**

The most distinct transcriptome profile and the majority of differentially expressed genes (DEGs) were identified in luminal epithelium (LE). Many DEGs were found only in the cell type-specific analysis. The functional classification of DEGs identified in specific endometrial cell types revealed various distinct functions and pathways. Genes related to immune activation, estrogen signaling pathway, embryo development, and cell proliferation were upregulated in LE of pregnant gilts. Genes involved in sterol biosynthetic and metabolic processes and extracellular matrix were upregulated in stroma. Genes associated with cell communication such as via exosomes and vesicles were found as differential in LE, glandular epithelium (GE), and stroma (S).

**Conclusions:**

This study revealed that conceptus signals induce different transcriptomic regulations in the endometrial compartments/cell types related to their specific function during recognition and establishment of pregnancy.

**Electronic supplementary material:**

The online version of this article (10.1186/s12864-018-4855-y) contains supplementary material, which is available to authorized users.

## Background

Uterine receptivity to conceptus implantation begins after hatching from the zona pellucida. The embryo pre-contacts with uterine luminal (LE) or superficial glandular (sGE) epithelia; later followed by apposition and adhesion between trophectoderm and uterine LE/sGE. Finally, limited or extensive endometrial invasion (depending on species and type of placenta) initiates placentation. These peri-implantation events are prerequisites for fetal and placental growth and development through the remainder of pregnancy [1, 2]. During the conception cycle, the porcine embryo undergoes the first cell divisions in the oviduct and arrives in the uterus in the 4 cells stage [3]. After hatching from the zona pellucida on Day 7, the porcine conceptus changes rapidly from spherical to tubular, and then filamentous form between Days 10 and 12 after ovulation [4]. This is accompanied by differentiation of trophoblast cells making the conceptus ready for implantation [5]. Maternal recognition and establishment of pregnancy in pigs requires a biphasic pattern of estrogen secretion by the conceptus, mainly 17β-estradiol (E_2_), increased between Days 11–12 and Day 15 and Days 25–30, respectively [6]. Previous studies also indicated that the porcine embryo exerts local effects on endometrial structures and functional parameters as well [7, 8]. The effects on the endometrial luminal epithelium structure, uterine blood flow [9], histotroph production and changes in prostaglandin F_2α_ (PGF2a) release are mainly caused by E_2_ through directly or indirectly mediating the release of other substances [10]. Influences on cellular organization, composition, and function derived from E_2_ effects is thought to facilitate in turn conceptus development and later plancentation [11]. The pro-inflammatory cytokine interleukin one beta 2 (IL1B2) which expression is dramatically increased only in expanding porcine conceptuses (Day 11–12), is proposed to contribute to embryo implantation [12]. Interleukin one beta 2 binds to the interleukin 1 receptor type 2 (IL1R2) on the epithelial surface which can activate the nuclear factor kappa-B (NFKB) system [13], followed by cytokines (tumor necrosis factor (TNFα), Interleukins (ILs), interleukin 6 family cytokine (LIF), and colony stimulating factor 2 (GMCSF)), chemokines (C-C motif chemokine ligand 5 (RANTES)), and the PTGS2 signaling pathway [14].

The embryo-endometrial cross-talk in the pig has evolved as a critical mechanism responding initially to the hormonal trigger in order to achieve receptivity and then amplify the decision under embryonic signals for pregnancy. Thus, the endometrium’s receptive ability and the prevention of luteolysis is very important during this critical period [5]. So far, a number of studies have been performed to analyze differential gene expression in porcine endometrium in response to embryonic signals starting from blastocyst stage [15] until placentation [16–19]. In our previous studies we investigated differential gene expression in porcine endometrium on Days 12 and 14 of pregnancy by the use of RNA-sequencing (RNA-seq) and identified numerous DEGs in response to the presence of conceptuses [18, 19]. Previous studies in the mouse conducted with Laser capture microdissection (LCM), a commonly used method to isolate different cell types from whole tissues or organs with high purity [20, 21], and microarrays indicated cell type-specific differences in transcriptome changes between LE and GE in response to pregnancy and revealed that different cell types act differentially and synergistically to permit blastocyst implantation [22]. Field et al. studied the immune-related gene expression in endometrial epithelial and stromal cells by LCM and microarrays, and found that the expression of factors involved in immunomodulation/endometrial remodeling was different between the epithelial and stromal compartments in a temporal manner [23]. Although the mouse is a good model to study the uterine transcriptome, the results can not be directly transferred to other species. Previously, Brooks et al. focused on the ovine cell-specific uterine transcriptome by the use of LCM and found a number of genes that contribute to cell proliferation, migration, attachment, and differentiation in ovine uterine LE [24]. Although a number of studies using LCM to dissect the endometrial cell types have been performed, none of them compared more than two cell types and did a comparison to the complete tissue sample. Since the knowledge about the cell-type specific gene expression is rather limited in porcine endometrium, the aim of the present study was to characterize the complex transcriptome changes in different endometrial cell types during the preimplantation period for improving our understanding of localization of endometrial gene expression regulation in context of recognition of pregnancy. To do this analysis in a transcriptome-wide manner, RNA-seq was performed for samples derived from luminal epithelium (LE), glandular epithelium (GE), and stroma (S) isolated by the use of LCM and compared to a corresponding data set for complete endometrial tissue samples.

## Methods

### Isolation of target cells

The animal experiment and sample collection is described in Samborski et al. [18]. Treatments of gilts were performed in accordance with the regulations of the local authorities (District Government of Upper Bavaria, Veterinary Office). The performed standard procedures/treatments in animal breeding were all in accordance with the International Guiding Principles for Biomedical Research Involving Animals, as proposed by the Society for the Study of Reproduction, with the European Convention on Animal Experimentation and with the German Animal Welfare Act. The animals were housed at farm facilities of the LMU Munich (Chair for Molecular Animal Breeding and Biotechnology) and slaughtered at the slaughterhouse of the Institute for Animal Breeding, Bavarian State Research Center for Agriculture, Poing, Germany.

A number of 8 prepuberal gilts were bought from a livestock trader (crossbreeds of German Landrace and Piétrain) and received a single injection of 750 IU PMSG (Intergonan®, MSD Animal Health Innovation GmbH, Schwabenheim, Germany) and 72 h later 750 IU hCG (Ovogest®, MSD Animal Health Innovation GmbH) to synchronize ovulation. Gilts of the “pregnant” group (*n* = 4) were inseminated twice (24 h and 36 h after hCG) with a standard dose of German Landrace semen whereas gilts of the “non-pregnant” control group (*n* = 4) were inseminated with the supernatant of centrifuged (10 min, 3000 rpm) semen of the same boar. Gilts were slaughtered on Day 12 after insemination. Endometrial samples (medial part of the uterine horns) were collected and snap-frozen in liquid nitrogen and stored at − 80° until preparation for LCM. Pregnancy was confirmed by the presence of filamentous conceptuses in the flush of the uterine horns. Briefly, frozen endometrium samples were cut in 10 μm thick sections with a Leica CM1950 clinical cryostat (Leica Biosystems, Germany), mounted onto membrane slides (MembraneSlide NF 1.0 PEN, Zeiss, Germany), and stained using a modified, rapid Cresyl violet staining protocol to identify LE, GE, and stromal cells. Briefly, the slides were first fixed with 70% ethanol (Sigma), and quickly washed in 50% ethanol. Cresyl violet was used to stain for 3 min, and then the stained slides were washed again with 50, 70, 100% ethanol, respectively (dip slides two/three times into each solution). Finally, the slides were dried at room temperature. All solutions were prepared with RNase-free water. The target cells from the sections were captured using a LCM Zeiss 200 M (inverse) microscope (Zeiss PALM Microsystems, Germany). When satisfactory cutting was achieved, the target tissue was lifted to the LCM cap (AdhesiveCap 200 clear, Zeiss, Germany) and 50 μl extraction buffer was used to incubate the LCM samples at 42 °C for 30 min to lyse the cells.

### Isolation of RNA, quality control, and RNA-sequencing

Total RNA was isolated from luminal epithelium, glandular epithelium, and stromal samples of each pig using the PicoPure RNA Isolation Kit (Applied Biosystems™, Vilnius, Lithuania) following the manufacturer’s instructions. Integrity and quantity of the RNA were assessed using the Agilent RNA 6000 Pico assay on the Agilent 2100 Bioanalyzer (Agilent Technologies, Waldbronn, Germany). The quality of the isolated total RNA extracted from LE, GE, and S were ranging from 6.6 to 9.4 (RNA integrity number, RIN). Most of the samples had a RIN around 8 were used to prepare a total of 24 RNA-seq libraries for biological replicates per group (*n* = 4 gilts) and cell type.

The Ovation SoLo Single Cell RNA-Seq System (NuGen Technologies, San Carlos, USA) was used for preparing RNA-Seq libraries starting from 500 pg of total RNA (corresponding to no more than 50 cells) according to the manufacturer’s recommendations. The number of PCR cycles for the first amplification step (determined by qPCR according to the manual) was between 15 and 20, for most of the samples 17. The 24 libraries were prepared from each individual sample for the three cell types of each replicate of both experimental groups. Then, all individual barcoded libraries were pooled for sequencing on two lanes of a single-read flow cell on an Illumina HiSeq 2500 instrument. Sequencing and demultiplexing were performed at the Functional Genomics Center Zurich (FGCZ).

### Bioinformatics and data analysis

The obtained sequence reads (Fastq files) were analyzed with a locally installed version of Galaxy [25]. The adapter sequence was clipped (for shorter fragments where sequencing runs into the adapter) and 5 bp were removed from the start of the reads. Sequences were mapped with Hisat2 (Sscrofa 11.1) from NCBI (ftp://ftp.ncbi.nih.gov/genomes/Sus_scrofa/GFF) and filtered by CPM cutoff after removing the PCR duplicates with NUGEN nudup based on the 8 random bases contained in the barcode adapter. The resulting read count table was used for statistical analysis in EdgeR to identify differentially expressed genes (DEGs) [26]. With a false discovery rate (FDR) of < 1% for LE and using its corresponding *p* value of 0.0021 as a cut-off for GE, stroma, and complete tissue, hierarchical cluster analysis was performed for DEGs in MultiExperiment Viewer (MeV). Gene ontology (GO) and pathway analysis was conducted by using the online tool ToppCluster and the Database for Annotation, Visualization, and Integrated Discovery (DAVID) [27]. The data set from complete endometrial tissue samples was analyzed with the same pipeline except the step for removal of PCR duplicates. Raw FASTQ files were deposited at National Center for Biotechnology Information (NCBI) Gene Expression Omnibus (GSE109539).

## Results

### Transcriptome sequencing of samples collected by LCM

Cells from uterine LE, GE, and S were isolated from endometria collected from Day 12 pregnant gilts (*n* = 4) and Day 12 nonpregnant cyclic gilts (control, *n* = 4) using LCM. Although the isolated stromal areas comprise a mix of fibroblasts and other cell types, mainly different immune cells, stroma (S) is in the following referred to as a “cell type”. A total of 458.1 million raw reads were obtained from the two experimental groups; 205.4 and 252.7 million in pregnant and control groups, respectively. After low-quality reads were filtered out and PCR duplicates were removed, a total of 259.3 million clean reads (116.1 in pregnant group, 143.2 million in control group) were selected for further analyses (Additional file 1: Table S1).

### Detected genes and DEGs in comparison of complete tissue and LCM samples

**Fig. 1:**
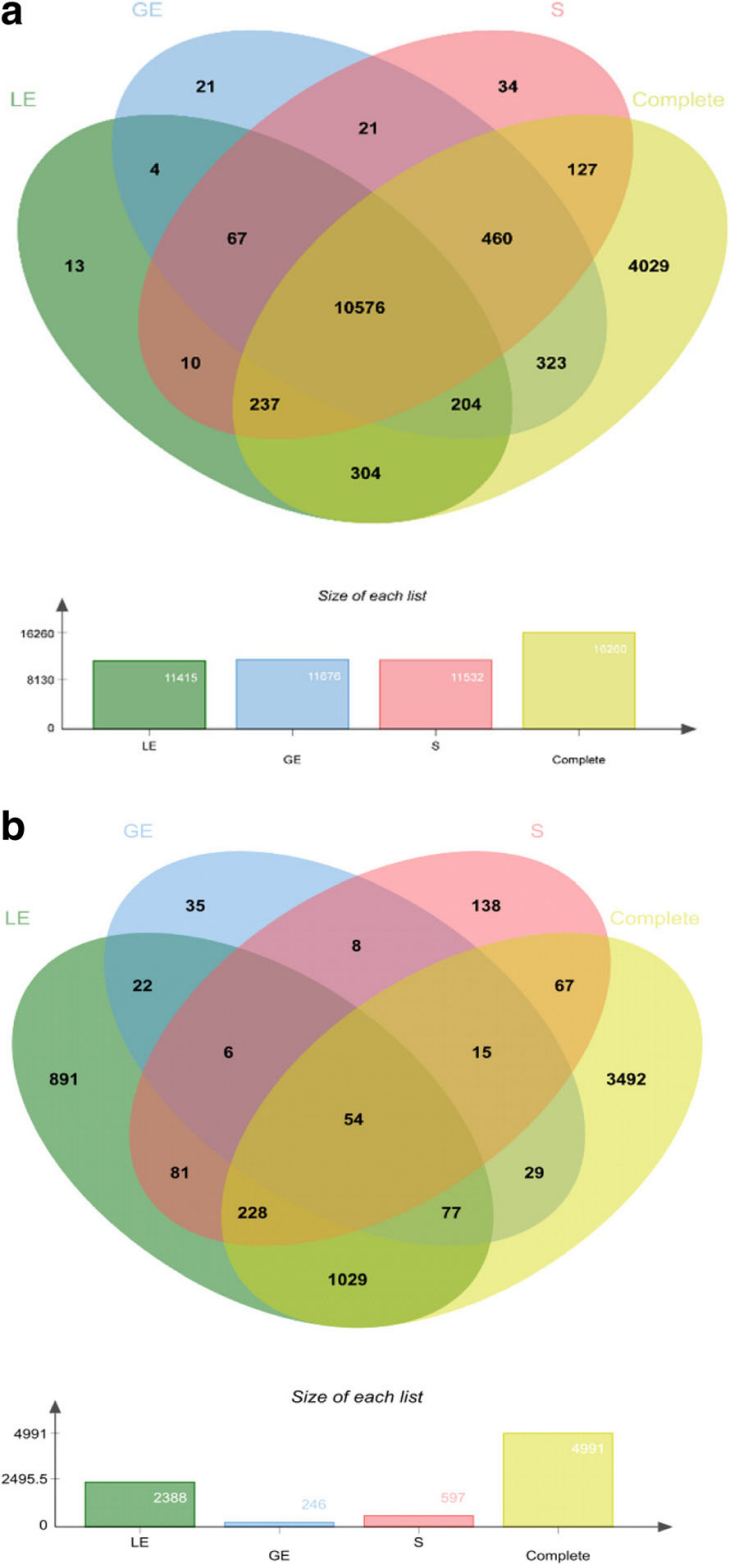
Venn diagram showing the overlaps of detectable genes (**a**) and differentially expressed genes (**b**). Green, luminal epithelium (LE); blue, glandular epithelium (GE); pink, stromal cells (S); yellow, complete tissue samples

The obtained LCM RNA-seq expression data were compared to data from complete endometrium tissue samples collected from Day 12 pregnant gilts and corresponding cyclic controls (raw data from Samborski et al. [18]). A total of 12,401 genes were detectable with the LCM method. For the individual endometrial compartments these were 11,415, 11,676, and 11,532 genes (LE, GE, and S, respectively). Clearly more genes (16260) were detectable in complete endometrial tissue samples. A high number of genes (10576) could be detected in the three LCM sample types and in complete tissue (Fig. 1a). Only 170 genes were detectable in the LCM samples but not in the complete sample. The numbers of genes putatively showing cell type-specific expression were 317, 344, and 161, for LE, GE, and stroma, respectively (expression detected in the corresponding cell type and for most of the genes also in complete tissue). Eleven solute carrier family members (*SLCs*) and 5 genes coding for transmembrane protein (TMEMs) were only detected in LE. In addition, 10 coiled-coil domain containing (*CCDC*) mRNAs were only found in GE. These genes detected only in one cell type were mostly expressed at rather low levels (data not shown).

The analysis of differential gene expression was performed between the pregnant and the nonpregnant group for each cell type and also for complete tissue samples. More DEGs were found for LE when compared with GE and S, i.e., 2388, 246, and 597 DEGs, respectively (Fig. 1b, Additional file 2: Table S2). Almost 5000 DEGs were obtained for the complete tissue samples in comparison of pregnant and nonpregnant gilts (Fig. 1b, Additional file 2:Table S2). Many of the DEGs, mainly for LE with 1920 DEGs, were differentially expressed (DE) in one cell type but not in the other cell types or in complete tissue (Fig. 1b, sector overlap LE with Complete and sector LE only). Also, for GE and S, many DEGs were specifically DE in these cell types (Fig. 1b). About half of all genes found to be DE in a cell type-specific way were not found as DE in the complete tissue sample.

### Unsupervised clustering of samples by multiple dimension scaling plots

**Fig. 2:**
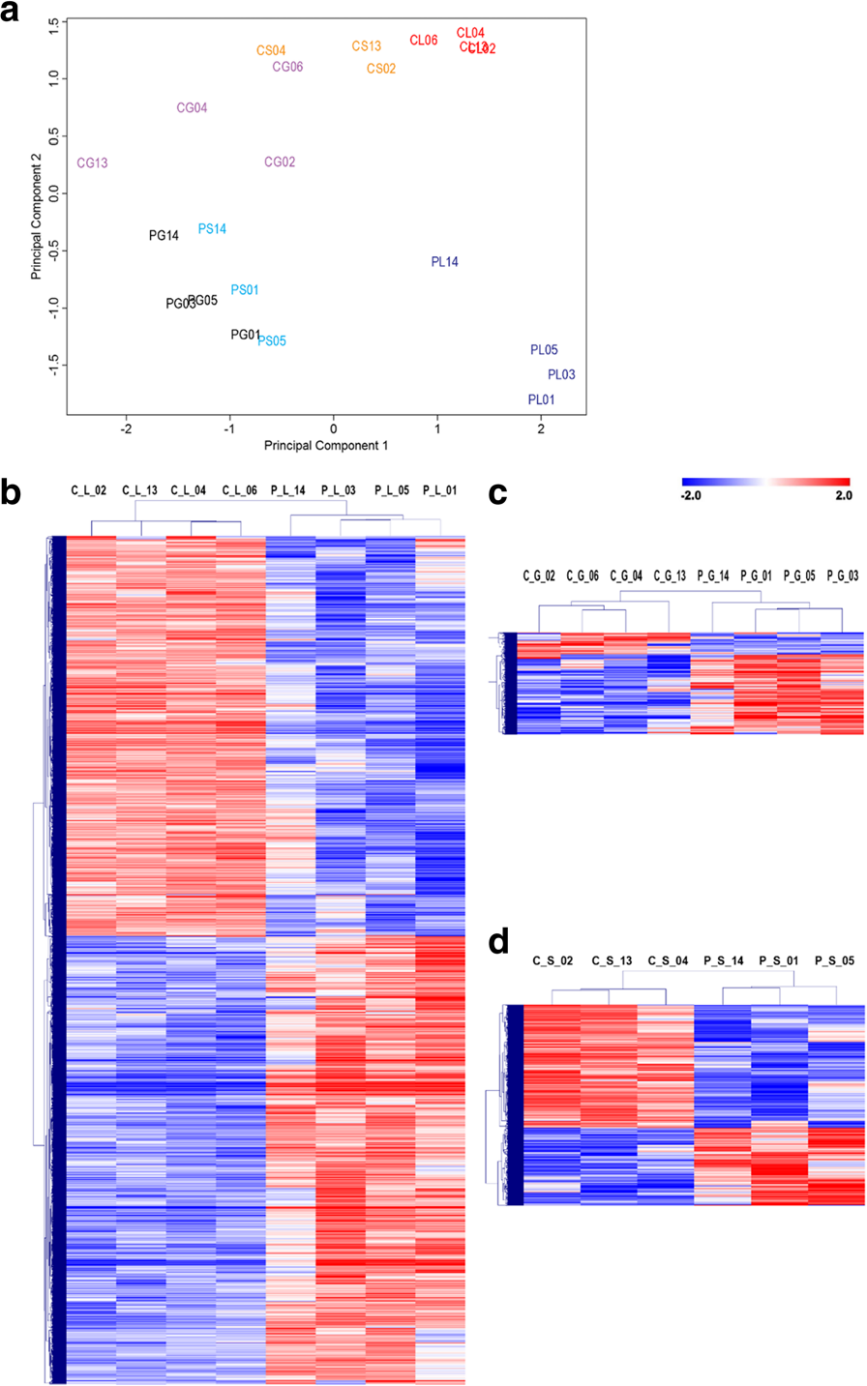
Unsupervised clustering of endometrial LCM samples. **a**. A multidimensional scaling plot (principal component analysis) for the 500 genes showing the highest pairwise fold changes between the samples in the dataset for LCM samples was performed in EdgeR. Samples from the same group are shown in the same color. **b-d** Hierarchical cluster analysis of differentially expressed genes identified for the luminal epithelium (**b**), glandular epithelium (**c**), and stroma (**d**). Mean-centered expression values (log2 counts per million of sample – mean of log2 counts per million of all samples) for the samples of the control and pregnant groups are shown for genes with significant differences in gene expression (FDR < 1%). Color scale is from − 2 (blue, lower than mean) to 2 (red, higher than mean). Each row represents 1 gene, each column 1 sample. The first letter of the sample IDs indicates the group (C or P: control or pregnant group) and the second the cell type (L, G, and S: luminal epithelium, glandular epithelium, and the stromal cells), followed by the ID of the animal, respectively

Multiple dimension scaling (MDS) plot analysis revealed for principal component 1 a grouping according to the individual cell types, particularly for the LE in the pregnant state (Fig. 2a). The GE and S samples derived from pregnant endometria were more similar to each other than the corresponding samples derived from the cyclic group. In the second dimension (principal component 2), a clear separation of pregnant and control samples was mainly found for LE, but also for GE and S (Fig. 2a). The lowest distance between the pregnant and cyclic control group was observed for GE corresponding to the lowest number of DEGs.

Furthermore, hierarchical cluster analysis was performed for the DEGs identified for each cell type (Figs. 2b, c, d). With respect to higher or lower expression in pregnant compared to cyclic endometrium, 1260 up-regulated and 1128 down-regulated, 193 up-regulated and 53 down-regulated, and 231 up-regulated and 366 down-regulated DEGs were identified in LE, GE and S, respectively (Additional file 2: Table S2).

### Functional category analysis of DEGs

**Fig. 3:**
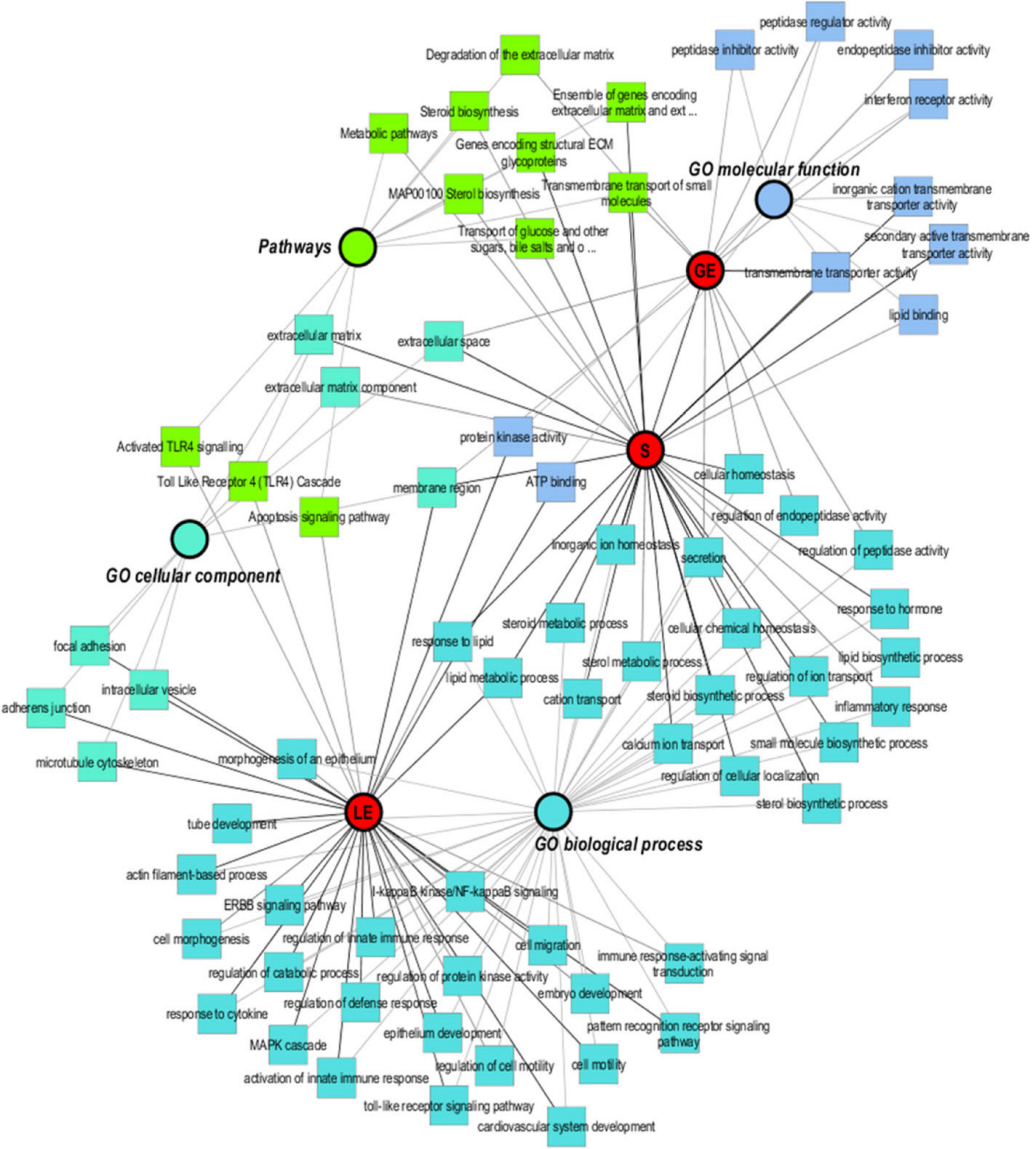
Gene Ontology (GO) functional classification network. All significant genes (human Entrez Gene IDs) in three cell types were used as input for the ToppCluster. The following databases were used, i.e. “biological process”, “cellular component”, “molecular function” and pathway. Finally, the data were uploaded in Cytoscape 3.4.0 to modify the network. Nodes were colored based on specificity: red nodes specific for luminal epithelium (LE), glandular epithelium (GE), and stromal cells (S); nodes for the three GO functions and pathway were in different colors

**Fig. 4:**
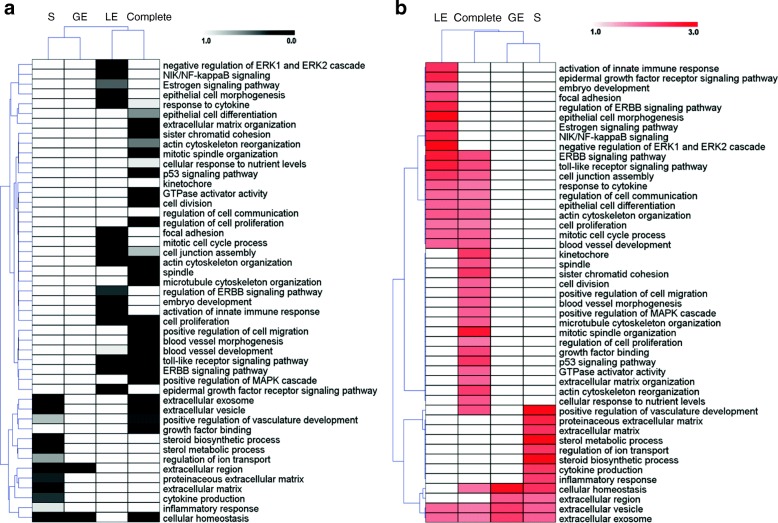
Comparison of significance of enrichment (false discovery rate, FDR) of selected functional categories and pathways between endometrial compartments and complete endometrium samples (**a**). Overrepresented functional categories and pathways were selected (FDR < 1%) from the results obtained for each cell type and the complete endometria. The FDR from 0 (black) to 1 (white) is shown for selected categories/pathways specific or in common for the studied cell types. Each row represents a function or pathway, each column a cell type or complete tissue (luminal epithelium (LE), glandular epithelium (GE), stromal cells (S), and complete tissue). Comparison of fold enrichment of selected functional categories and pathways between endometrial compartments and complete endometrium samples (**b**). Overrepresented functional categories and pathways were selected (FDR < 1%) from the results obtained for each cell type and the complete endometria. The fold enrichment from 1 (white, no enrichment) to 3 (red, 3-fold enrichment) is shown for selected categories/pathways specific or in common for the studied cell types. Each row represents a function or pathway, each column 1 cell type or complete tissue (luminal epithelium (LE), glandular epithelium (GE), stromal cells (S), and complete tissue)

An overview of the network illustrating functional classification of the obtained DEGs is shown in Fig. 3. All DEGs of LCM were analyzed with ToppCluster tool for Gene Ontology (GO) and pathway analysis. Selected specifically enriched functional categories are shown for biological process (BP), molecular function (MF), cellular component (CC), and pathways for LE, GE, and S, respectively. The majority of overrepresented categories such as adhesion junction, cell motility, embryo development and MAPK cascade were obtained for LE. Cellular homeostasis, metal transport, and steroid metabolic process were found for stroma. Peptidase inhibitor activity and membrane transporter activity were enriched for GE samples. A systematic comparison of overrepresented GO categories and KEGG pathways between the endometrial cell types and complete endometria was performed with the DAVID tool. At a FDR of 1%, 47 overrepresented functional categories and KEGG pathways were found in total. For selected categories, the FDR and the fold enrichment obtained for the three cell types and complete endometria was used to generate a heatmap in order to illustrate specific and common overrepresented categories and pathways (Figs. 4a and b). Based on this comparison, DEGs were enriched for the functional categories “extracellular exosome” and “extracellular vesicle” in all three cell types as well as complete tissue. Specific enrichment of DEGs in LE was found for categories related to cell communication and signaling (e.g. estrogen signaling), immune response, epithelium development, cell proliferation, cytoskeleton, cell junction and migration. In contrast, DEGs found in stroma were enriched for functional categories and pathways involved in sterol and steroid biosynthesis and metabolism, inflammatory response, extracellular matrix, and ion transport. Particular enrichment for DEGs in complete endometria was identified for categories related to cell division and blood vessel morphogenesis. For GE, the number of enriched terms and pathways was generally very low due to the low number of DEGs. More detailed information about the comparison of overrepresented categories and pathways and the assigned DEGs for specific cell types and complete tissue is shown in Additional file 3: Table S3.

### Genes differentially expressed in specific cell types or in complete tissue

**Fig. 5:**
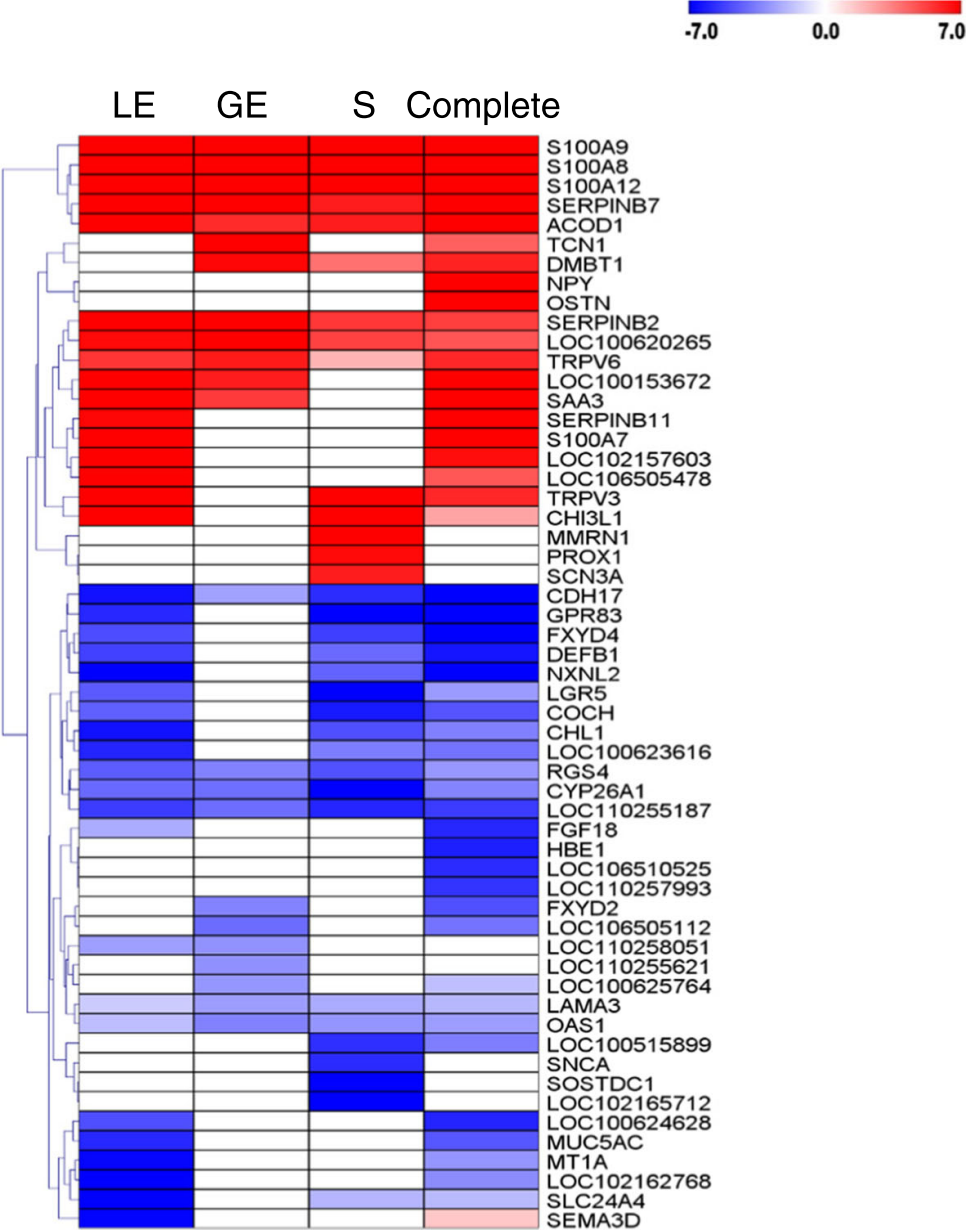
Heatmap for the log2 fold changes (pregnant/control) of the top 10 differentially expressed genes of each gene expression comparison. The scale is from log2 FC -7 (blue, downregulated in pregnant samples) to log2 FC 7 (red, upregulated in pregnant samples). Each row represents 1 gene, each column 1 cell type or tissue (luminal epithelium (LE), glandular epithelium (GE), stromal cells (S), and complete tissue)

First, the log2 fold changes (FC) (pregnant/control) of the top 10 up- and downregulated DEGs for each cell type and complete endometrial tissue were compared (Fig. 5, Additional file 4: Table S4). Genes such as multimerin 1 (*MMRN1*), prospero homeobox 1 (*PROX1*), sodium voltage-gated channel alpha subunit 3 (*SCN3A*), synuclein alpha (*SNCA*), sclerostin domain containing 1 (*SOSTDC1*), *LOC102165712* were only DE in stroma. The genes neuropeptide Y (*NPY*), osteocrin (*OSTN*), hemoglobin subunit epsilon 1 (*HBE1*), *LOC106510525*, *LOC110257993* could only be found as DE in complete tissue. Some members of the S100 calcium binding protein A family were highly upregulated in all three cell types, except S100 calcium binding protein A7 (*S100A7*) which was specifically upregulated in LE. Furthermore, serpin family B members 2 and 7 were highly upregulated in all cell types whereas serpin family B member 11 (*SERPINB11*) in LE only (not detectable in GE and stroma).

Genes were defined as specifically DE if they were contained in the Venn diagram in the not overlapping sectors of each cell type and the sector for the overlap of one cell type with the DEGs found in the complete tissue samples (Fig. 1b). Furthermore, genes were analyzed that were DE only in the complete tissue samples, which may well contain genes DE in cell types not present in the LCM samples. Functional annotation results (overrepresentation analysis) for genes only DE in certain cell types or complete tissue are shown in Additional file 5: Table S5. The main overrepresented functional categories for the genes specifically upregulated in LE were related to “phosphorus metabolic process”, “ATP binding”, “signal transduction”, “cell adhesion/junction”, “cell migration”, “extracellular vesicle”, “amide and peptide biosynthetic process”, “protein transport”, “cytoskeleton organization”, “immune response”, and “cell cycle”. The genes specifically downregulated in LE were involved in “metal ion binding”, “cell morphogenesis”, “epithelium development”, “microtubule cytoskeleton”, and “DNA repair”. Only a few overrepresented functional categories were found for GE-specific DEGs, “endoplasmic reticulum” and “extracellular vesicle” for upregulated genes. Specific DEGs for stromal regions were enriched for “ion transport”, “cytoskeleton organization”, “cell morphogenesis”, and “blood vessel development” (upregulated genes) and “cellular lipid metabolic process”, “steroid biosynthetic process”, “endoplasmic reticulum”, and “metal ion binding” (downregulated genes). Highly significant enrichment was obtained for many functional categories in the analysis of upregulated genes specific for complete tissue. The main processes and functions were “cell cycle”, “cell proliferation”, “vasculature or blood vessel development”, “cell motility”, “cell death”, “proteolysis”, “signal transduction”, “cell adhesion”, “leukocyte activation”, and “extracellular vesicle”. For the downregulated genes, specific for complete tissue, overrepresentation was found for “mitochondrion”, “steroid dehydrogenase activity”, “ciliary part”, “cilium organization”, and a variety of metabolic processes.

### DEGs involved in estrogen signaling, prostaglandin metabolism and signaling, and other selected pathways

A number of 249 DEGs assigned to a selection of particularly interesting pathways and processes, e.g., estrogen signaling, steroid hormone biosynthesis, prostaglandin (PG) metabolism, signaling and transport, interleukin-1 (IL1) and interferon (IFN) type I signaling are shown in Additional file 6: Table S6. A total number of 122 DEGs including 81 up- and 41 downregulated genes were found as DE in LE. For the GE, 16 were up- and 3 downregulated. In addition, a number of 20 genes were upregulated and 22 genes were downregulated in stroma. For complete endometrial samples, 117 assigned genes were upregulated and 69 were downregulated. For some of the pathways, functionally related genes showed distinct localization of gene expression regulation and in part the same direction of regulation (i.e., collective up- or downregulation).

### Comparison of the LCM RNA-seq results to data from real-time RT-PCR and published localization studies

Overall, the overlap of the DEGs between the LCM samples and the complete tissue samples (dataset from Samborski et al. [18]) showed the reliability of the LCM RNA-seq data. Furthermore, a comparison to results of quantitative PCR for 25 selected genes from Samborski et al. [18] and other studies was performed (Table 1). Furthermore, the localization of endometrial gene expression of 20 selected genes observed by LCM RNA-seq was compared to results from in situ hybridization (ISH) and immunohistochemistry (IHC) (Table 2). Both comparisons showed very good agreement and in addition the superior sensitivity in comparison to ISH and IHC.

**Table 1:** Comparison of RNA-seq and qPCR data

Ssc gene symbol	Ssc Entrez Gene ID	Hsa gene symbol	Hsa Entrez Gene ID	Hsa gene description	log2 FC P/C						P-value*						
					RNA-seq				qPCR		RNA-seq				qPCR		
					LE	GE	S	Complete	D12	D14	LE	GE	S	Complete	D12	D14	PubMed ID
*ABCC1*	733,619	*ABCC1*	4363	ATP binding cassette subfamily C member 1	3.5	1.4	1.1	2.4	1.6	–	< 0.001	0.224	0.321	< 0.001	< 0.01	–	27764917
*LOC100738425*	100,738,425	*ABCC4*	10,257	multidrug resistance-associated protein 4	nd	2.9	nd	2.2	2.1	–	–	0.019	–	0.001	< 0.001	–	24695625
*ABCC9*	100,127,449	*ABCC9*	10,060	ATP binding cassette subfamily C member 9	1.2	0.0	0.6	2.2	1.8	–	< 0.001	0.999	0.844	< 0.001	< 0.05	–	27764917
AKR1B1	396,816	AKR1B1	231	aldo-keto reductase family 1 member B	5.2	5.2	2.2	5.1	5.8	–	< 0.001	< 0.001	0.060	< 0.001	< 0.001	–	24695626
*BAALC*	100,170,128	*BAALC*	79,870	brain and acute leukemia, cytoplasmic	nd	nd	nd	−5.0	−5.8	–	–	–	–	< 0.001	< 0.001	–	24174570
*LOC100157834*	100,157,834	*CDH17*	1015	cadherin 17, LI cadherin (liver-intestine)	−6.5	−2.6	−5.8	−8.3	−6.3	–	< 0.001	0.001	< 0.001	< 0.001	< 0.001	–	24174570
*CLDN1*	100,625,166	*CLDN1*	9076	claudin 1	5.0	nd	4.2	3.4	1.8	–	< 0.001	–	< 0.001	< 0.001	0.002	–	25123632
*PBD-2*	404,699	*DEFB1*	1672	defensin, beta 1	−5.2	−1.7	−4.1	−6.4	−5.9	−3.4	< 0.001	0.015	< 0.001	< 0.001	< 0.001	0.010	24174570
*FGF9*	396,717	*FGF9*	2254	fibroblast growth factor 9 (glia-activating factor)	2.2	nd	nd	3.0	3.7	3.0	< 0.001	–	–	< 0.001	< 0.001	< 0.001	24174570
*FGFR3*	100,514,115	*FGFR3*	2261	fibroblast growth factor receptor 3	−1.2	−0.1	−1.1	−2.3	–	− 0.6	< 0.001	0.991	0.066	< 0.001	–	0.048	20393170
*LOC100511475*	100,511,475	*FXYD4*	53,828	FXYD domain containing ion transport regulator 4	−4.9	nd	−5.2	−7.4	−7.5	–	< 0.001	–	< 0.001	< 0.001	< 0.001	–	24174570
*LOC100513220*	100,513,220	*GPR83*	10,888	G protein-coupled receptor 83	−5.9	nd	−7.5	− 7.8	−4.0	–	< 0.001	–	< 0.001	< 0.001	< 0.001	–	24174570
*HPGD*	100,156,186	*HPGD*	3248	hydroxyprostaglandin dehydrogenase 15-(NAD)	−3.8	−1.4	−3.6	−3.0	−2.7	–	< 0.001	0.005	< 0.001	< 0.001	< 0.01	–	24695626
*LOC100739374*	100,739,374	*IL24*	11,009	interleukin 24	−2.8	−1.4	− 2.7	−4.6	−5.2	−5.3	< 0.001	0.254	< 0.001	< 0.001	0.024	0.033	24174570
*IRF1*	396,611	*IRF1*	3659	interferon regulatory factor 1	0.6	−0.1	0.4	0.4	0.5	2.9	0.115	0.890	0.397	0.087	0.188	0.003	24174570
*IRG1*	100,524,951	*IRG1*	730,249	immunoresponsive 1 homolog (mouse)	10.3	5.8	6.2	9.9	8.0	–	< 0.001	0.000	< 0.001	< 0.001	< 0.001	–	24174570
*OSTN*	100,049,691	*OSTN*	344,901	osteocrin	nd	nd	nd	10.3	12.4	–	–	–	–	< 0.001	< 0.001	–	24174570
*S100A9*	100,127,489	*S100A9*	6280	S100 calcium binding protein A9	12.4	11.5	11.3	10.4	10.6	9.2	< 0.001	< 0.001	< 0.001	< 0.001	< 0.001	< 0.001	24174570
*SERPINB7*	100,152,588	*SERPINB7*	8710	serpin peptidase inhibitor, clade B, member 7	10.4	7.9	6.2	10.3	8.9	5.4	< 0.001	< 0.001	< 0.001	< 0.001	< 0.001	0.018	24174570
*SLCO2A1*	100,144,510	*SLCO2A1*	6578	solute carrier organic anion transporter family member 2A1	1.0	0.4	2.3	0.5	3.0	–	0.011	0.960	< 0.001	0.053	< 0.001	–	24695625
*SLCO4C1*	100,737,557	*SLCO4C1*	353,189	solute carrier organic anion transporter family member 4C1	1.6	0.2	1.3	2.3	2.6	–	0.007	0.978	0.174	< 0.001	< 0.05	–	27764917
*SLCO5A1*	100,157,250	*SLCO5A1*	81,796	solute carrier organic anion transporter family member 5A1	8.4	4.9	5.9	6.4	9.2	–	< 0.001	< 0.001	< 0.001	< 0.001	< 0.05	–	27764917
*SPP1*	397,087	*SPP1*	6696	secreted phosphoprotein 1	4.1	1.6	3.2	2.4	2.5	3.2	< 0.001	0.005	< 0.001	< 0.001	0.008	0.029	24174570
*LOC100738308*	100,738,308	*STAT1*	6772	signal transducer and activator of transcription 1, 91 kDa	1.6	0.2	0.8	1.4	1.2	2.3	< 0.001	0.562	0.066	< 0.001	0.001	< 0.001	24174570
*STC1*	100,125,345	*STC1*	6781	stanniocalcin 1	−2.9	−1.0	−2.9	1.9	2.5	2.0	< 0.001	0.384	< 0.001	< 0.001	0.101	0.017	24174570

Ssc: *Sus scrofa*; Hsa: *Homo sapiens*; P/C: pregnant vs. control; *for RNA-seq adjusted *p*-values; nd: not detectable

**Table 2:** Comparison of LCM RNA-seq results for endometrial localization of gene expression to results from in situ hybridization and immunohistochemistry

Gene	LE	GE	S	Complete	Other studies	Technique	PubMed ID
*AKR1B1*	5.2	5.2	2.2	5.1	LE days 12/13 P	ISH	17640989, 24695626
*HPGD*	−3.8	−1.4	−3.6	−3.0	expression in LE D12C	ISH	24695626
*EGFR*	−0.7	d	d	−1.0	low abundance in LE/GE D12	ISH	24012778
*FGF7*	2.3	1.7	d	2.1	expression in LE/GE D12P	ISH	10819782
*FGF9*	2.2	nd	nd	3.0	expression in GE D14P	IHC	20393170
*FGFR2*	d	d	d	d	expression in LE/GE D12	ISH	28395342
*ABCC1*	3.5	d	d	2.4	expression in LE/GE D12P (weak)	ISH	27764917
*LOC100738425* (*ABCC4*)	nd	2.9	nd	2.2	expression in LE/GE D12P	ISH/IHC	24695625
*ABCC9*	1.2	d	d	2.2	expression in LE/GE D12P (weak)	ISH	27764917
*SLC24A4*	−8.2	d	−2.0	−1.9	nd in LE D12P	ISH	24472379
*SLCO2A1*	1.0	d	2.3	d	expression in LE/BV D12P	ISH	24695625
*SLCO4C1*	1.6	d	d	2.3	nd, ISH too weak, qPCR D12P up	ISH	27764917
*SLCO5A1*	8.4	4.9	5.9	6.4	nd, ISH too weak, qPCR only D12 P	ISH	27764917
*GJA1*	2.0	d	1.3	1.3	stroma, BV	IHC	9669753
*GJB1*	nd	nd	nd	−1.3	nd in LE	IHC	9669753
*GJB2*	nd	nd	nd	nd	nd in LE	IHC	9669753
*IRF2*	1.3	d	d	1.0	cycle: low all cell types, from D12P up in LE	ISH	17475929
*S100G*	2.9	d	d	3.6	expression in LE D12P	ISH	19641180
*STAT2*	−0.8	d	d	d	cycle: low in all cell types, after D12P: up in stroma	ISH	17475929
*TRPV6*	5.6	6.3	2.1	5.9	strong expression in LE, weak in GE D12P	ISH	19641180

Values are log2 FC; d: expression detected; nd: expression not detectable; LE: luminal epithelium; GE: glandular epithelium; BV: blood vessels; P: pregnant; C: cyclic; ISH: in situ hybridization; IHC: immunohistochemistry

## Discussion

The present study implemented laser capture microdissection (LCM) to isolate endometrial LE, GE, and stroma from the porcine uterus with the aim of uncovering important cell-specific gene expression regulation, which is masked in the transcriptome analysis of whole endometrium samples due to the complex cell type composition of this mucosal tissue. Overall, the obtained results and the clear sample clustering based on the RNA-seq data generated by this approach agreed very well with our previous studies utilizing RNA-seq of complete tissue samples and the confirmation of selected DEGs by real-time RT-PCR [18, 19]. Furthermore, a comparison to published results for localization of expression of selected genes in porcine endometrium revealed good agreement and showed higher sensitivity of the LCM RNA-seq approach compared to ISH and IHC. In comparison to the data derived from complete endometrial tissue, less detectable genes were found for the certain cell types/compartments. This may be attributed to the fact that the endometrial tissue has a complex and dynamic cellular composition, which includes LE, GE, fibroblasts, endothelial cells, pericytes, and various immune cells. However, the number of more than 11,500 detectable genes obtained from the analysis of the LCM samples is in the expected range of transcribed genes for the analysis of an individual cell type.

The results for the LCM samples revealed that there was a clear difference in gene expression between non-pregnant and pregnant samples, particularly for the LE. This indicates that the main effects of the conceptus signaling on endometrial gene expression are localized to the luminal epithelium. Furthermore, more than half of the genes DE in LCM samples were not found as differential in complete tissue, showing that analysis of the whole endometrium is partially missing cell type-specific gene regulation. Some genes not appearing as DE in whole endometria can even be expressed both in LE and GE or stroma but with opposite expression regulation, which eventually results in similar expression between pregnant and non-pregnant state in complete tissue.

### Genes preferentially differentially expressed in endometrial cell types are related to specific functions

For the genes specifically DE in LE overrepresented functional categories were mainly related to signaling, immune functions, and cell adhesion. This is probably reflecting the various responses to the signals derived from the elongating conceptus mainly affecting the LE. Genes upregulated in LE and assigned, e.g., to “protein phosphorylation” and other categories related to signaling could be induced by conceptus-derived interleukin 1 beta which has been suggested to play an important role in development of the LE by stimulating cell proliferation via activation of the ERK1/2 and P38 MAPK cell signaling cascades [28]. Furthermore, endometrial- and/or conceptus derived epidermal growth factor (EGF) has been shown to stimulate phosphorylation of ERK1/2 MAPK in porcine LE cells and proposed to regulate development of the peri-implantation uterine LE at the fetal-maternal interface [29].

For stroma, DEGs, related to “vasculature development”, “ion transport”, “cell development”, were overrepresented. Angiopoietin-2 (*ANGPT2*) mRNA was detectable in all three cell types but only upregulated in stroma and complete endometria in this study. ANGPTs comprise a second key group of vascular regulators in the endometrium with interactions with the vascular endothelial growth factor (VEGF) system and appear to play a major role in the regulation of blood vessel growth, maturation, and regression [30, 31]. In human endometrium, ANGPT2 expression was mainly localized to the glandular epithelium and endothelium [32]. While only 11 genes upregulated in stroma (3.8-fold enrichment) were assigned to “vasculature development”, 116 genes upregulated in complete endometria (2.4-fold enrichment) were assigned, showing that most of the genes involved in this process are regulated in endometrial regions rich in blood vessels which were not collected for the stroma samples. These genes included several angiogenesis-related genes such as *VEGFC*, *VEGFD*, and VEGF receptors 1, 2, and 3 as well as angiopoietin receptors, and a number of endothelial cell markers. The high enrichment scores obtained for “blood vessel development” and related categories is in concordance with the finding that conceptus estrogen can increase endometrial vascular permeability [33].

The functional category “ion transmembrane transport” showed higher enrichment for genes upregulated in stroma compared to genes upregulated in LE of pregnant group. However, many more genes were assigned to this category for genes DE in LE. In LE and stroma calcium transporter genes had lower expression in samples from pregnant gilts, particularly solute carrier family 24 member 4 (*SLC24A4*) with a log2 FC of − 8.2. This is in agreement with a recent study where *SLC24A4* mRNA was detectable in LE cells during the estrous cycle by in situ hybridization, but undetectable during early pregnancy including Day 12 [34]. In addition, the mRNAs for the calcium ion channel protein TRPV6 and the intracellular calcium-regulatory molecule S100G were upregulated in all three cell types but highest in LE of pregnant gilts which is in agreement with results from quantitative PCR and localization by ISH of a previous study [35].

Altogether, the analysis of genes specifically DE in endometrial compartments showed differential local responses to the conceptus-derived signals. Selected functional categories important for maternal recognition of pregnancy (MRP) and establishment of pregnancy are discussed in the following.

### Genes involved in cell communication and endometrial remodeling

In the present study, genes involved in extracellular exosomes/vesicles (EVs) were overrepresented in DEGs of LE, GE, and stroma. In sheep, EVs that carry genetic materials (mRNAs, proteins and miRNAs) were found in uterine luminal fluid (ULF), indicating its potential role in the conceptus-endometrial interaction [36]. The overrepresentation of this theme indicates a similar importance of EVs for embryo-maternal interactions as suggested for humans [37].

In addition to cell-cell adhesion (see below), cell junctions play also an important role in cell-to-cell communication. Genes involved in cell junction assembly, e.g., members of the connexin family were enriched for DEGs in LE. A number of connexins was upregulated, namely gap junction protein alpha (*GJA1*, *GJA4*, and *GJA5*), beta (*GJB3*, *GJB4*, and *GJB5*), and gamma (*GJC1*). Connexin 43 (*GJA1*) was expressed in all three cell types and upregulated in LE and stroma. Expression of *GJA4* and *GJB4* was only detectable in complete endometrium samples and *GJA5* only in GE. Expression and upregulation of *GJB3* was found in all three cell types with highest expression in LE whereas *GJB5* mRNA was only detected in LE of Day 12 pregnant gilts. In comparison to findings of connexin expression in human and rodent endometrium and placenta, there are some similarities, but also distinct differences probably related to the non-invasive type of placentation in the pig. In humans as well as in rodents, expression of connexin 26 (*GJB2*) is suppressed in LE and connexin 43 (*GJA1*) is suppressed in stromal cells during the receptive phase and expression of *GJB2* is induced by the presence of a blastocyst and by proinflammatory factors such as PGF2a and IL1B [38]. In contrast, in porcine endometrium, expression of *GJB2* mRNA was not detectable on Day 12 of pregnancy and the estrous cycle, respectively and *GJA1* mRNA was upregulated on Day 12. Expression of connexins in porcine endometrium could be controlled by a combination of conceptus estrogens and IL1B2 and PGs. So far, differential expression of *GJA4*, *GJA5*, *GJB3*, *GJB4*, and *GJB5* has been described only in trophoblast cells of human and/or mouse placenta [39], but not in endometrial cells. In addition, several genes of the tight junction protein family (claudins) were found as DE, *CLDN1* upregulated in LE and stroma, *CLDN3* and *CLDN4* upregulated only in LE, and *CLDN8* downregulated in LE and stroma. The distinct expression pattern of connexins and claudins in porcine endometrium suggests an important role in the interaction of the different functional compartments of the endometrium and with the conceptus.

The functional term “epithelial cell morphogenesis” was significantly enriched for LE. In this context, a number of growth factor and growth factor receptor genes have been found as differentially expressed in porcine endometrium during the preimplantation phase [40]. Fibroblast growth factor 7 (*FGF7*) mRNA was upregulated in LE and GE but not in stromal regions in agreement with the results of a previous study [41]. FGF7 secreted to the uterine lumen has been suggested to have autocrine effects on the endometrium as well as paracrine effects on the conceptus trophectoderm [42]. The mRNA for the high-affinity receptor for FGF7, *FGFR2* was expressed in all three endometrial cell types at high levels, supporting FGF7 could have autocrine effects on the endometrium. The mRNA of another fibroblast growth factor, *FGF9* was specifically upregulated in LE in agreement with the results in one of our previous studies [43]. Upregulation of *FGF9* expression was also found during the phase of maternal recognition of pregnancy in the mare [44].

In human endometrium, stroma-derived insulin-like growth factors (IGFs) are implicated in growth regulation of epithelial cells [45]. In porcine endometrium, mRNAs for *IGF1* and *IGF2* as well as their receptors were not DE on Day 12 but detected in all three endometrial cell types, with high expression levels for *IGF1*, *IGFR1*, and *IGFR2*. A number of mRNAs for IGF binding proteins (*IGFBP2*, *IGFBP3*, *IGFBP4*, *IGFBP5*, *IGFBP6*, *IGFBP7*) were also detected, but only *IGFBP2* was found as upregulated in complete endometria, suggesting upregulation in blood vessel regions. Another growth factor gene, *EGF* and its receptor (*EGFR*) were DE in porcine endometrium, *EGF* downregulated in LE and stroma and *EGFR* downregulated in LE. A recent study in cyclic and ovariectomized gilts revealed downregulation of endometrial *EGFR* expression in ovariectomized estradiol-treated gilts while *EGF* remained unchanged [46]. Localization of expression in porcine endometrium by ISH revealed low abundance of *EGFR* mRNA in LE and GE between days 9 and 15 of the estrous cycle and days 9 and 12 of pregnancy [47].

The transforming growth factor beta (TGFB) signaling pathway has been identified as an important modulator of many endometrial functions during the sexual cycle, the implantation phase and placentation [48]. Most of the genes assigned to the TGFB signaling pathway were found as DE in complete endometria and some in stroma, such as bone morphogenetic protein 2 and 4 (*BMP2*, *BMP4*), suggesting a role in vascular remodeling. *TGFB1* and *TGFB2* were expressed in LE, GE, and stroma and were upregulated in complete endometrial samples, *TGFB3* was identified as upregulated in stroma only. Inhibin beta B subunit (*INHBB*) mRNA was upregulated in LE and stroma.

**Fig. 6:**
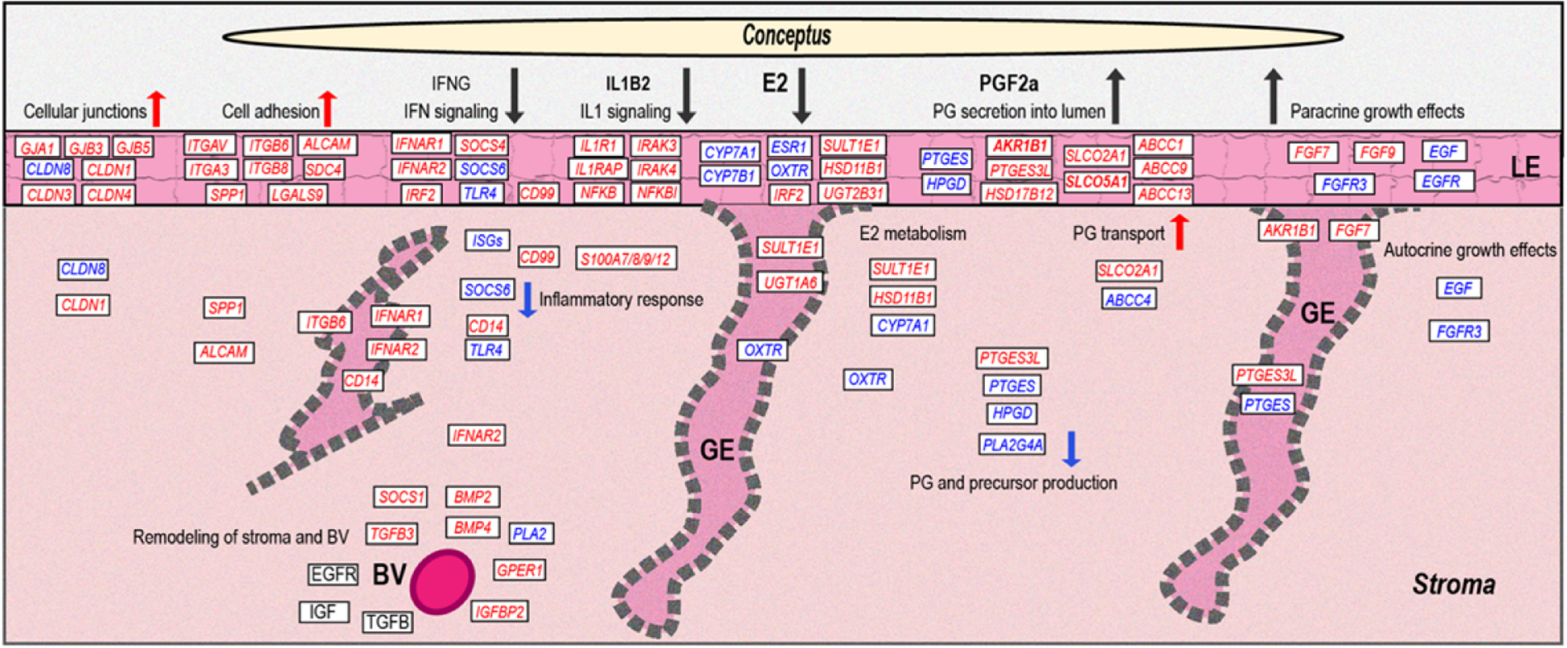
Summary of the main findings of the study. This schematic overview is based on the results of the present study of endometrial localization of differential gene expression. Genes highlighted in red and blue color were found as up- and downregulated, respectively when comparing pregnant to nonpregnant sows

In summary, intercellular communication is regulated by cellular junctions in a species-specific manner in context of the strictly non-invasive type of implantation in the pig. Several growth factor systems are regulated in the endometrium. The FGF system seems to be involved in autocrine and paracrine effects on the epithelium and conceptus, respectively. Based on the localization of gene expression regulation of the IGF, EGFR, and TGFB systems, suggests a role mainly in remodeling of stromal regions and blood vessels (overview in Fig. 6).

### Genes related to cell adhesion

Members of the integrin family were reported to be critical for endometrium-conceptus communication and implantation, as well as cell adhesion cascades [49]. Two integrin alpha genes (*ITGA1* and *ITGAV*) and six integrin beta genes (*ITGB1*, *ITGB2*, *ITGB3*, *ITGB5*, *ITGB6*, and *ITGB8*) showed higher mRNA concentrations in complete endometrium on Day 12 of pregnancy. The results from the LCM samples revealed four integrin genes, *ITGAV*, *ITGA3*, *ITGB6*, and *ITGB8* as upregulated in LE, *ITGB6* in addition upregulated in GE. In line with the mRNA expression, ITGA3 protein was localized to the uterine epithelium during early pregnancy in the pig [50]. The genes for *ITGA1*, *ITGB1*, *ITGB2*, *ITGB3*, *ITGB5, ITGB6*, and *ITGB8* were only DE in the complete tissue samples. Based on the known functions of these integrins, it is likely that they are DE in B or T cells and/or blood vessels.

The expression of osteopontin (also known as Secreted Phosphoprotein 1, SPP1), a secreted ECM protein that binds to a variety of cell surface integrins, has been shown to increase markedly in LE during the peri-implantation period in pigs [51]. In our study, *SPP1* mRNA was upregulated in LE and also in stroma. It is known that expression of the integrin heterodimer alpha(v)beta(6), which is binding to SPP1, on the endometrium and the trophoblast is necessary for conceptus attachment as the ability of trophectoderm cells to bind to OPN decreased after knocking down *ITGAV* [52].

A number of further genes assigned to the GO category “cell-cell adhesion” were upregulated in LE. Galectin 9 (*LGALS9*) mRNA was specifically upregulated in LE, and a similar expression profile was also reported in bovine endometrium [53]. Popovici et al. characterized the cells that express *LGALS9* in human endometrium, and found that *LGALS9* mRNA was significantly upregulated during early pregnancy in epithelial cells, whereas it was not expressed in stromal cells [54]. Additionally, syndecan 4 (*SDC4*) was upregulated in LE. In mice, *SDC4* was found as upregulated in LE and GE, and also in endometrial fibroblasts [55]. Another mRNA for a cell adhesion molecule, activated leukocyte cell adhesion molecule (*ALCAM*) was upregulated in LE and stroma. The results of a previous study showing that ALCAM is over-expressed in human endometrial cell during the implantation period [56] and expression of ALCAM on human endometrial epithelial cells and blastocysts [57] suggests that the ALCAM-ALCAM cell adhesion system is probably involved in the interaction between the embryo and maternal endometrium in humans and the pig.

The observed expression of genes known as involved in cell adhesion, shows that the attachment of the conceptus trophectoderm to the LE following after day 13 of pregnancy is already initiated on Day 12 (overview in Fig. 6).

### Genes related to immune response

In context of conceptus-endometrium interactions in the pig, a number of signaling molecules involved in regulation of immune response have been described [58]. The pig conceptus-specific IL1B2 plays an important role during conceptus elongation and establishment of pregnancy through its effects on the uterine luminal epithelium [59, 60]. In the context of IL1B signaling, interleukin 1 receptor type 1 (*IL1R1*), interleukin 1 receptor accessory protein (*IL1RAP*), and interleukin 1 receptor associated kinases 3 and 4 (*IRAK3*, *IRAK4*) were upregulated in LE in the present study. In agreement with upregulation of *IL1R1* in LE, a previous report showed that secretion of IL1B2 from the conceptus is associated with increased endometrial expression of *IL1R1* [61]. In general, interleukin 1 beta is one of the major signaling molecules in the NFKB signaling pathway. Many members of this central immune response signaling pathway were found to be differentially expressed in LE, e.g., mRNAs for a number of NF-kappa B subunits (*NFKB1*, *NFKB2*, *REL*, *RELB*) were upregulated as well as NFKB inhibitors (*NFKBIA, NFKBIB, NFKBIE*). A recent study revealed that compared to IL1B1, the extent of NFKB activation and related gene expression was lower in endometrium treated with IL1B2 in the pig [59]. The lower NFKB activation observed for the IL1B2 secretion from the conceptus could be associated with the simultaneous upregulation of *NFKBIA, NFKBIB,* and *NFKBIE*.

In addition to the IL1 and NFKB signaling pathway, a number of genes involved in the interferon type I signaling pathway (WP585) were obtained as DE. Members of the signal transducer and activator of transcription family (*STAT1*, *STAT4*, *STAT5A*, *STAT5B*) were mainly found as upregulated in LE, except *STAT2* which was downregulated in LE. Protein inhibitor of activated STAT 1 (*PIAS1*) was also found as upregulated but only in the complete endometria. Furthermore, a number of genes of the type I IFN signaling pathway were differentially expressed. Interferon alpha and beta receptor subunit 1 (*IFNAR1*) and *IFNAR2* were both upregulated in LE and GE, and all three LCM samples, respectively. In a recent study, upregulation of *IFNAR1* and *IFNAR2* mRNAs was also found on Day 12 of pregnancy in porcine endometrium and positive regulation by IL1B for *IFNAR1* mRNA and by IL1B and E2 for *IFNAR2* mRNA [62]. In contrast, the expression of interferon regulatory factors *IRF3*, *IRF4*, *IRF7*, and *IRF9* was lower in samples from Day 12 pregnant gilts, *IRF3*, *IRF4*, *IRF7* in complete endometria and *IRF9* in LE. Furthermore, a number of typical IFN-stimulated genes (*IFI6*, *IFI30*, *IFI44*, *IFI44L*, *IFIT1*, *IFIT2*, *IFIT3*, *IFITM1*, *IFITM3*, *MX1*, 2′-5′-oligoadenylate synthetases (*OAS1*, *OAS2*), and radical S-adenosyl methionine domain containing 2 (*RSAD2*) in LE and/or stroma) which are upregulated by IFNT in ruminants, showed lower expression in samples derived from Day 12 pregnant gilts. These findings are in line with the results from our previous studies [18, 19, 63] which showed that the endometrial response to conceptus signals has very distinct characteristics in comparison of Days 12 and 14 of pregnancy and a typical response to type I IFNs can only be observed on Day 14. In agreement with a previous study [64], increased expression of *IRF2* mRNA in LE was found in pregnant endometrium supporting upregulation by conceptus-derived E2.

However, in the pig, interferon gamma (IFNG) is the major trophoblast-derived IFN on Day 12 of pregnancy, which is synthesized between Days 12 and 20 during the gestation period [65]. Interferon gamma receptor 2 (*IFNGR2*) was upregulated in all three cell types of the pregnant endometrium in this study. Interferon gamma plays a crucial role in innate immune responses [66], and its functions are achieved via receptor-mediated signals that lead to changes in the transcription of hundreds of genes [67]. But since a strong inflammatory response would have negative effects on establishment of pregnancy, IFNG effects have to be regulated. Suppressor of cytokine signaling 1 (SOCS1) has been identified as a specific negative regulator of IFNG effects [68]. *SOCS1* mRNA was upregulated in complete endometria. In addition, *SOCS4* was found as upregulated in LE, whereas *SOCS6* was downregulated in LE and stroma.

Members of the S100 calcium binding protein A gene family have been found as involved in innate immunity [69]. Particularly *S100A7*, *S100A8*, *S100A9*, and *S100A12* were highly upregulated in pregnant sows (log2 FC up 8–12). Members of this protein family are in addition important for the modulation of the inflammatory response [70].

Many CD molecules genes (e.g. *CD14*, *CD34*, *CD36*, *CD40*, *CD46*, *CD58*, *CD93,* and *CD99*) were found as upregulated in complete tissue. In LCM samples, *CD14* was found as upregulated in GE and stroma and was not detectable in LE. The CD14 molecule is an accessory molecule of toll like receptor 4 (TLR4) which is important for innate immune responses to bacterial and other microbial structures [71]. The mRNA for *TLR4* was downregulated in LE and stroma. The expression patterns of *CD14* and *TLR4* suggest a specific and local modulation of proinflammatory effects in the porcine during early pregnancy. In addition, *CD99* was found as upregulated in LE and stroma, which has a major regulatory function in early T-cells [72]. Inducible T-cell costimulator ligand (*ICOSLG*) playing a role in regulation of endometrial T-cells [73] was upregulated in LE which could also be involved in modulation of the maternal immune system.

The expression patterns of genes involved in immune response reflects the effects of conceptus- and endometrium-derived signaling molecules. Overall, the involved genes and their spatial regulation suggests a tight control of the maternal immune response to support conceptus growth and to avoid negative inflammatory effects (overview in Fig. 6).

### Genes involved in estrogen signaling and metabolism

Estrogen receptor alpha (*ESR1*) expression has been studied in endometrium of gilts and sows during the estrous cycle and early pregnancy [74–76]. No differences in *ESR1* mRNA expression was found between cyclic and pregnant endometrium on Days 11–12. Here, we found three-fold lower mRNA concentrations in LE of samples from pregnant gilts. The mRNA encoding the membrane-bound G protein-coupled estrogen receptor 1 (*GPER1*) was also downregulated, but only in the complete tissue samples. The fact that GPER1 was only found as DE in complete endometrium samples suggests regulation near blood vessel regions. Downregulation of *GPER1* mRNA could result from downregulation of *ESR1* since it has been shown that *GPER1* is regulated by nuclear ESR1 and progesterone receptors [77].

The functional categories “sterol biosynthetic process” and “steroid metabolic process” were specifically overrepresented for the DEGs found in stroma. Genes encoding UDP-glucuronosyltransferases (UGTs) were upregulated, UDP glucuronosyltransferase 1 family, polypeptide A6 (*UGT1A6*) in GE, UDP-glucuronosyltransferase 2B31 (*UGT2B31*) in LE, and UDP-glucuronosyltransferase 2B31-like (*LOC100515741*) in complete endometria. As UGT enzymes are involved in glucuronidation of E2 [78], this indicates increased UGT-mediated estrogen metabolism on Day 12 of pregnancy. Cytochrome P450 family members *CYP1B1*, *CYP7A1*, *CYP7B1* which are involved in E2 synthesis and metabolism [79, 80], were also DE on Day 12 of pregnancy. For example, *CYP7A1* regulating cholesterol metabolism [81] was downregulated in LE and stroma, on the other side, *CYP7B1* being related to steroid metabolism [82] was downregulated in LE. Sulfotransferase family 1E, estrogen-preferring, member 1 (*SULT1E1*) involved in steroid synthesis and metabolism has been found as upregulated in porcine endometrium during Days 15 and 16 of pregnancy [83]. We found that *SULT1E1* is already upregulated on Day 12 in LE, GE, and stroma. Overall, the results for genes involved in E2 metabolism showed downregulation of genes involved in E2 synthesis, while genes probably mediating inactivation of E2 (sulfatases, UGTs) were upregulated in the endometrium on Day 12 of pregnancy (see overview in Fig. 6).

### Genes involved in prostaglandin metabolism and signaling

Members of the phospholipase A2 family (PLA2s), key enzymes for the release of PG precursor molecules from the plasma membrane [84], were downregulated mainly in complete endometrial tissue samples, except *PLA2G4A* which was localized to stromal areas. The downregulation of PLA2 genes in complete endometria could indicate regulation associated with immune cells or cells of blood vessels. In a recent study, a trend for downregulation of *PLA2G4A* has been found on Day 12 of pregnancy whereas no difference was found on Day 15 [85]. The aldo-keto reductase family 1 member B (AKR1B1) is an aldose reductase enzyme that is secreted into the extracellular spaces where it functions in the synthesis of PGs, specifically PGF2a in the endometrium [86]. Strong upregulation of *AKR1B1* mRNA was found only in LE and GE with strongest expression in LE, while prostaglandin-endoperoxide synthase 2 (*PTGS2*) was downregulated only in stroma. This leads us to hypothesize that AKR1B1, together with PTGS2 maybe important enzymes involved in the change of endocrine to exocrine secretion of PGF2a in porcine endometrium. Furthermore, the mRNA for *HSD17B12* was upregulated in LE. Its known function in conversion of estrone to estradiol is in contrast to the observed downregulation of other genes involved in E2 synthesis, but HSD17B12 is also known to function in arachidonic acid metabolism thereby providing precursors for prostaglandin synthesis [87]. Together with the localization of upregulation in LE this suggests a role in PG synthesis on Day 12 of pregnancy in porcine endometrium. The mRNA for prostaglandin E synthase (*PTGES*) was found as downregulated in LE. In addition, *PTGES2*, *PTGES3*, and *PTGES3L* were also expressed in all three cell types (*PTGES2* not in GE). The mRNA of *PTGES3L* was more than 4-fold upregulated in LE of pregnant endometrium. However, the expression of *PTGES3* mRNA was clearly higher compared to the mRNAs of the other PGE2 synthases. Similar to the findings in a recent study [85], *PTGES3* mRNA was slightly higher in LE on Day 12 of pregnancy but did not reach the significance threshold (adjusted *P*-value 0.042 in LE, 0.021 in complete tissue).

The biological effects of PGs are also controlled by PG transport, e.g., mediated by specific transporters such as transmembrane transporters ATP-binding cassette, subfamily C, member 4 (ABCC4) and solute carrier organic anion transport family, member 2A1 (SLCO2A1). In porcine endometrium, *ABCC4* and *SLCO2A1* expression has been found as upregulated on Day 12 of pregnancy in LE and GE, and LE and blood vessels, respectively [88]. In our study, *ABCC4* was downregulated and *SLCO2A1* was upregulated in LCM samples from stromal areas. A closer look at the RNA-seq data showed that *ABCC4* is expressed in all three cell types. Likewise, *SLCO2A1* mRNA was detectable at similar expression levels in all three cell types. In GE, another gene (*LOC100738425*) similar to human *ABCC4* was expressed and showed a 7.5-fold increase in samples from Day 12 pregnant gilts. Altogether, the obtained results revealed a very complex pattern of regulation for PG synthesizing, metabolizing and transporting proteins, as well as PG receptors. Basically, the results from our endometrial LCM RNA-seq analysis suggest a specific upregulation of the PGF synthase AKR1B1 in LE and GE, specific uregulation of PG transporters in the LE, local upregulation of PGE synthases, and a general downregulation of PG precursor synthesis in other regions of the endometrium as the molecular mechanism for the switch from endocrine to exocrine PGF2a secretion and the regulation of PGE2 production, which needs further comprehensive functional studies at the protein level (for an overview see Fig. 6).

## Conclusions

In summary, this study comprehensively determined differential gene expression in the endometrial compartments/cell types LE, GE, and stroma of porcine endometrium during the preimplantation period. In comparison to previous studies, the localization of transcriptomic changes in response to conceptus signals was addressed and used to draw a global picture of molecular pathways involved in establishment and maintenance of pregnancy in the pig. The findings of this study will serve as a basis for in-depth investigations of cell type-specific molecular pathways in porcine during the phase of maternal recognition of pregnancy.

## Additional files

Additional file 1: **Table S1.** Raw data statistic of RNA-seq (DOCX 15 kb)

Additional file 2: **Table S2.** Differentially expressed genes in luminal epithelium, glandular epithelium, stroma, and complete tissue. (XLSX 1826 kb)

Additional file 3: **Table S3.** Selected function found in luminal epithelium, glandular epithelium, stroma, and complete tissue. (XLSX 58 kb)

Additional file 4: **Table S4.** Top 10 differentially expressed genes in luminal epithelium (LE), glandular epithelium (GE), and stromal cells (S). (DOCX 20 kb)

Additional file 5: **Table S5.** Overrepresentation analysis for genes only differentially expressed in three cell types and complete tissue. (XLSX 687 kb)

Additional file 6: **Table S6.** DEGs involved in estrogen signaling, prostaglandin metabolism and signaling, and other selected pathways. (XLSX 117 kb)

## Abbreviations

ABCC4: ATP-binding cassette, subfamily C, member 4
ABI3BP: ABI family member 3 binding protein
AKR1B1: Aldo-keto reductase family 1 member B
ALCAM: Activated leukocyte cell adhesion molecule
ALDH2: Aldehyde dehydrogenase 2 family
ANGPT2: Angiopoietin-2
B4GALNT2: Beta-1,4-N-acetyl-galactosaminyltransferase 2
BMP: Bone morphogenetic protein
CALM: Calcium/calmodulin genes
CCDC: Coiled-coil domain containing
CDs: CD molecules
CENPK: Centromere protein K
CYP1B: Cytochrome P450 family members
DE: Differentially expressed
DEGs: Differentially expressed genes
ECM: Extracellular matrix
EGF: Epidermal growth factor
EGFR: Epidermal growth factor receptor
ESR1: Estrogen receptor alpha
ETV1: ETS variant 1
EVs: Extracellular exosomes/vesicles
FGF: Fibroblast growth factor
FRAS1: Extracellular matrix gene 1
FRK: Fyn related Src family tyrosine kinase
GE: Glandular epithelium
GJA: Gap junction protein alpha
GJB: Gap junction protein beta
GJC: Gap junction protein gamma
GMCSF: colony stimulating factor 2
GO: Gene ontology
GPER1: G protein-coupled estrogen receptor 1
HBE1: Hemoglobin subunit epsilon 1
HSD11B1: Hydroxysteroid 11-beta dehydrogenase 1
ICOSLG: Inducible T-cell costimulator ligand
IGF: Insulin-like growth factor
IGFBP: Insulin-like growth factor binding protein
IL1B2: Interleukin one beta 2
IL1R: Interleukin 1 receptor
IL1RAP: Interleukin 1 receptor accessory protein
INHBB: Inhibin beta B subunit
IRAK: Interleukin 1 receptor associated kinase
IRF2BP2: Interferon regulatory factor 2 binding protein 2
ITGs: Integrin genes
LAP: Latency-associated peptide
LCM: Laser capture microdissection
LE: Luminal epithelium
LFA-1: Lymphocyte function-associated antigen-1
LGALS9: Galectin 9
LIF: Interleukin 6 family cytokine
LLC: Large latent complex
LTBP: Transforming growth factor beta binding protein
MMRN1: Multimerin 1
MTR: Methyltetrahydrofolate-homocysteine methyltransferase
NCBI: National Center for Biotechnology Information
NFKB: Nuclear factor kappa-B
NPY: Neuropeptide Y
NR3C1: Glucocorticoid receptor
OAS: 2′-5′-oligoadenylate synthetase
OPN: Osteopontin
OSTN: Osteocrin
PG: Prostaglandin
PGF2a: Prostaglandin F2α
PIAS1: Protein inhibitor of activated STAT 1
PLA2s: Phospholipase A2 family
PROX1: Prospero homeobox 1
PTGES: Prostaglandin E synthase
PTGS2: Prostaglandin-endoperoxide synthase 2
RANTES: C-C motif chemokine ligand 5
RIN: RNA integrity number
RNA-Seq: RNA-sequencing
ROCK: Rho associated coiled-coil containing protein kinase
RORB: RAR related orphan receptor B
RSAD2: Radical S-adenosyl methionine domain containing 2
S100A: S100 calcium binding protein A
SALL2: Spalt like transcription factor 2
SCN3A: Sodium voltage-gated channel alpha subunit 3
SDC4: Syndecan 4
SERPINB11: Serpin family B member 11
SLC24A4: Solute carrier family 24 member 4
SLCO2A1: Solute carrier organic anion transport family, member 2A1
SLCs: Solute carrier family members
SNCA: Synuclein alpha
SOCS: Suppressor of cytokine signaling
SOSTDC1: Sclerostin domain containing 1
STAT: Signal transducer and activator of transcription family
SULT1E1: Sulfotransferase family 1E, estrogen-preferring, member 1
TGFB: Transforming growth factor beta
TLR4: Toll like receptor 4
TMEMs: Transmembrane protein
TNFα: Tumor necrosis factor
UGTs: UDP-glucuronosyltransferases
ULF: Uterine luminal fluid
VEGF: Vascular endothelial growth factor
