# Supplementary material for: Cell type-specific analysis of transcriptome changes in the porcine endometrium on Day 12 of pregnancy
Source: BMC Genomics. 2018 Jun 14;19:459. doi: 10.1186/s12864-018-4855-y (PMC6000939; doi:10.1186/s12864-018-4855-y)
Supplement: Supplementary file 1 — Table S1. Raw data statistic of RNA-seq (DOCX 15 kb) [file 12864_2018_4855_MOESM1_ESM.docx]

**Table S1. Raw data statistic of RNA-seq**

| **Sample** | **Total Raw Reads** | **Total Clean Reads** | **N percentage** | **GC percentage** |
| --- | --- | --- | --- | --- |
| C_G_02 | 23093437 | 18189370 | 0.00% | 43% |
| C_G_04 | 21973569 | 16963031 | 0.00% | 40% |
| C_G_06 | 21010355 | 16600773 | 0.00% | 42% |
| C_G_13 | 23595458 | 18606417 | 0.00% | 42% |
| C_L_02 | 33598003 | 25313849 | 0.00% | 46% |
| C_L_04 | 25631715 | 19716496 | 0.00% | 46% |
| C_L_06 | 15700508 | 12590934 | 0.00% | 43% |
| C_L_13 | 24795123 | 19944657 | 0.00% | 44% |
| C_S_02 | 21756884 | 16897682 | 0.00% | 41% |
| C_S_04 | 27182875 | 21232330 | 0.00% | 44% |
| C_S_13 | 14398251 | 11560930 | 0.00% | 42% |
| P_G_01 | 23726733 | 18027642 | 0.00% | 47% |
| P_G_03 | 17369665 | 13614506 | 0.00% | 46% |
| P_G_05 | 18232663 | 14435990 | 0.00% | 42% |
| P_G_14 | 25584390 | 20538411 | 0.00% | 40% |
| P_L_01 | 8208256 | 6402599 | 0.00% | 47% |
| P_L_03 | 16392427 | 12204894 | 0.00% | 45% |
| P_L_05 | 18874412 | 14956157 | 0.00% | 41% |
| P_L_14 | 17162245 | 13704076 | 0.00% | 42% |
| P_S_01 | 17884905 | 12084128 | 0.00% | 47% |
| P_S_05 | 19075961 | 15394218 | 0.00% | 41% |
| P_S_14 | 22872036 | 18223336 | 0.00% | 41% |
