# Supplementary material for: Cell type-specific analysis of transcriptome changes in the porcine endometrium on Day 12 of pregnancy
Source: BMC Genomics. 2018 Jun 14;19:459. doi: 10.1186/s12864-018-4855-y (PMC6000939; doi:10.1186/s12864-018-4855-y)
Supplement: Supplementary file 4 — Table S4. Top 10 differentially expressed genes in luminal epithelium (LE), glandular epithelium (GE), and stromal cells (S). (DOCX 20 kb) [file 12864_2018_4855_MOESM4_ESM.docx]

Supplemental Table S4. Top 10 differentially expressed genes in luminal epithelium (LE), glandular epithelium (GE), and stromal cells (S).

| Tissue | Ssc-ID | Ssc-Symbol | Description | Hsa-ID | Hsa-Symbol | Log2FC P/C | PValue |
| --- | --- | --- | --- | --- | --- | --- | --- |
| LE | 100127489 | *S100A9* | S100 calcium binding protein A9 | 6280 | *S100A9* | 12.42 | 0.00 |
|  | 100127488 | *S100A8* | S100 calcium binding protein A8 | 6279 | *S100A8* | 10.81 | 0.00 |
|  | 100152588 | *SERPINB7* | serpin family B member 7 | 8710 | *SERPINB7* | 10.41 | 0.00 |
|  | 100524951 | *ACOD1* | aconitate decarboxylase 1 | 730249 | *ACOD1* | 10.28 | 0.00 |
|  | 100301483 | *S100A12* | S100 calcium binding protein A12 | 6283 | *S100A12* | 10.08 | 0.00 |
|  | 100515051 | *TRPV3* | transient receptor potential cation channel subfamily V member 3 | 162514 | *TRPV3* | 9.92 | 0.00 |
|  | 102157603 | *LOC102157603* | uncharacterized LOC102157603 |  |  | 9.87 | 0.00 |
|  | 733603 | *SAA3* | serum amyloid A-3 protein |  |  | 9.60 | 0.00 |
|  | 100519286 | *SERPINB2* | serpin family B member 2 | 5055 | *SERPINB2* | 9.04 | 0.00 |
|  | 106505478 | *LOC106505478* | uncharacterized LOC106505478 |  |  | 8.64 | 0.00 |
|  | 110259210 | *MUC5AC* | mucin 5AC, oligomeric mucus/gel-forming | 4586 | *MUC5AC* | -5.90 | 0.00 |
|  | 100513220 | *GPR83* | G protein-coupled receptor 83 | 10888 | *GPR83* | -5.90 | 0.00 |
|  | 100623616 | *LOC100623616* | arylacetamide deacetylase-like |  |  | -5.99 | 0.00 |
|  | 100157834 | *CDH17* | cadherin 17 | 1015 | *CDH17* | -6.47 | 0.00 |
|  | 100511780 | *CHL1* | cell adhesion molecule L1 like | 10752 | *CHL1* | -6.49 | 0.00 |
|  | 397417 | *MT1A* | metallothionein 1A | 4489 | *MT1A* | -6.77 | 0.00 |
|  | 100523915 | *SEMA3D* | semaphorin 3D | 223117 | *SEMA3D* | -6.90 | 0.00 |
|  | 100739001 | *NXNL2* | nucleoredoxin-like 2 | 158046 | *NXNL2* | -7.70 | 0.00 |
|  | 102162768 | *LOC102162768* | uncharacterized LOC102162768 |  |  | -7.88 | 0.00 |
|  | 100156914 | *SLC24A4* | solute carrier family 24 member 4 | 123041 | *SLC24A4* | -8.20 | 0.00 |
| GE | 100127489 | *S100A9* | S100 calcium binding protein A9 | 6280 | *S100A9* | 11.48 | 0.00 |
|  | 100127488 | *S100A8* | S100 calcium binding protein A8 | 6279 | *S100A8* | 10.13 | 0.00 |
|  | 100519286 | *SERPINB2* | serpin family B member 2 | 5055 | *SERPINB2* | 8.80 | 0.00 |
|  | 100301483 | *S100A12* | S100 calcium binding protein A12 | 6283 | *S100A12* | 8.65 | 0.00 |
|  | 100152588 | *SERPINB7* | serpin family B member 7 | 8710 | *SERPINB7* | 7.91 | 0.00 |
|  | 396873 | *TCN1* | transcobalamin 1 | 6947 | *TCN1* | 7.60 | 0.00 |
|  | 100620265 | *LOC100620265* | cytochrome P450 4F22 | 126410 | *CYP4F22* | 7.05 | 0.00 |
|  | 751862 | *DMBT1* | deleted in malignant brain tumors 1 | 1755 | *DMBT1* | 6.81 | 0.00 |
|  | 100171400 | *TRPV6* | transient receptor potential cation channel subfamily V member 6 | 55503 | *TRPV6* | 6.26 | 0.00 |
|  | 100153672 | *LOC100153672* | olfactory receptor 13F1-like |  |  | 6.21 | 0.00 |
|  | 100511642 | *LAMA3* | laminin subunit alpha 3 | 3909 | *LAMA3* | -2.67 | 0.00 |
|  | 100625764 | *LOC100625764* | leucine carboxyl methyltransferase 1-like |  |  | -2.87 | 0.00 |
|  | 110258051 | *LOC110258051* | hematological and neurological expressed 1-like protein pseudogene |  |  | -2.99 | 0.00 |
|  | 110255621 | *LOC110255621* | uncharacterized LOC110255621 |  |  | -3.03 | 0.00 |
|  | 541593 | *FXYD2* | FXYD domain containing ion transport regulator 2 | 486 | *FXYD2* | -3.40 | 0.00 |
|  | 100157437 | *RGS4* | regulator of G protein signaling 4 | 5999 | *RGS4* | -3.41 | 0.00 |
|  | 397570 | *OAS1* | 2'-5'-oligoadenylate synthetase 1 | 4938 | *OAS1* | -3.42 | 0.00 |
|  | 100124374 | *CYP26A1* | cytochrome P450, family 26, subfamily A, polypeptide 1 | 1592 | *CYP26A1* | -3.97 | 0.00 |
|  | 110255187 | *LOC110255187* | uncharacterized LOC110255187 |  |  | -3.99 | 0.00 |
|  | 106505112 | *LOC106505112* | uncharacterized LOC106505112 |  |  | -4.03 | 0.00 |
| S | 100127489 | *S100A9* | S100 calcium binding protein A9 | 6280 | *S100A9* | 11.28 | 0.00 |
|  | 100516922 | *MMRN1* | multimerin 1 | 22915 | *MMRN1* | 10.03 | 0.00 |
|  | 100515051 | *TRPV3* | transient receptor potential cation channel subfamily V member 3 | 162514 | *TRPV3* | 8.17 | 0.00 |
|  | 100127488 | *S100A8* | S100 calcium binding protein A8 | 6279 | *S100A8* | 7.96 | 0.00 |
|  | 396865 | *CHI3L1* | chitinase 3 like 1 | 1116 | *CHI3L1* | 7.93 | 0.00 |
|  | 100301483 | *S100A12* | S100 calcium binding protein A12 | 6283 | *S100A12* | 7.33 | 0.00 |
|  | 100169702 | *PROX1* | prospero homeobox 1 | 5629 | *PROX1* | 6.68 | 0.00 |
|  | 100524951 | *ACOD1* | aconitate decarboxylase 1 | 730249 | *ACOD1* | 6.22 | 0.00 |
|  | 100625056 | *SCN3A* | sodium voltage-gated channel alpha subunit 3 | 6328 | *SCN3A* | 6.22 | 0.00 |
|  | 100152588 | *SERPINB7* | serpin family B member 7 | 8710 | *SERPINB7* | 6.20 | 0.00 |
|  | 100515899 | *LOC100515899* | uncharacterized LOC100515899 |  |  | -5.66 | 0.00 |
|  | 100157834 | *CDH17* | cadherin 17 | 1015 | *CDH17* | -5.76 | 0.00 |
|  | 641350 | *SNCA* | synuclein alpha | 6622 | *SNCA* | -5.78 | 0.00 |
|  | 110255187 | *LOC110255187* | uncharacterized LOC110255187 |  |  | -5.94 | 0.00 |
|  | 100624179 | *COCH* | cochlin | 1690 | *COCH* | -6.30 | 0.00 |
|  | 100736999 | *SOSTDC1* | sclerostin domain containing 1 | 25928 | *SOSTDC1* | -7.11 | 0.00 |
|  | 100124374 | *CYP26A1* | cytochrome P450, family 26, subfamily A, polypeptide 1 | 1592 | *CYP26A1* | -7.23 | 0.00 |
|  | 102165712 | *LOC102165712* | uncharacterized LOC102165712 |  |  | -7.27 | 0.00 |
|  | 100151994 | *LGR5* | leucine rich repeat containing G protein-coupled receptor 5 | 8549 | *LGR5* | -7.44 | 0.00 |
|  | 100513220 | *GPR83* | G protein-coupled receptor 83 | 10888 | *GPR83* | -7.48 | 0.00 |
| Complete | 100127488 | *S100A8* | S100 calcium binding protein A8 | 6279 | *S100A8* | 10.45 | 0.00 |
|  | 100127489 | *S100A9* | S100 calcium binding protein A9 | 6280 | *S100A9* | 10.41 | 0.00 |
|  | 100152588 | *SERPINB7* | serpin family B member 7 | 8710 | *SERPINB7* | 10.34 | 0.00 |
|  | 100049691 | *OSTN* | osteocrin | 344901 | *OSTN* | 10.29 | 0.00 |
|  | 100524951 | *ACOD1* | aconitate decarboxylase 1 | 730249 | *ACOD1* | 9.93 | 0.00 |
|  | 397304 | *NPY* | neuropeptide Y | 4852 | *NPY* | 8.85 | 0.00 |
|  | 100301483 | *S100A12* | S100 calcium binding protein A12 | 6283 | *S100A12* | 8.67 | 0.00 |
|  | 100153672 | *LOC100153672* | olfactory receptor 13F1-like |  |  | 8.55 | 0.00 |
|  | 100156623 | *SERPINB11* | serpin family B member 11 | 89778 | *SERPINB11* | 8.43 | 0.00 |
|  | 100144623 | *S100A7* | S100 calcium binding protein A7 | 6278 | *S100A7* | 8.39 | 0.00 |
|  | 110257993 | *LOC110257993* | uncharacterized LOC110257993 |  |  | -5.55 | 0.00 |
|  | 106510525 | *LOC106510525* | uncharacterized LOC106510525 |  |  | -5.77 | 0.00 |
|  | 100525013 | *FGF18* | fibroblast growth factor 18 | 8817 | *FGF18* | -5.92 | 0.00 |
|  | 100624628 | *LOC100624628* | lithostathine-like |  |  | -5.99 | 0.00 |
|  | 407067 | *HBE1* | hemoglobin subunit epsilon 1 | 3046 | *HBE1* | -6.13 | 0.00 |
|  | 404699 | *DEFB1* | defensin beta 1 | 1672 | *DEFB1* | -6.37 | 0.00 |
|  | 100511475 | *FXYD4* | FXYD domain containing ion transport regulator 4 | 53828 | *FXYD4* | -7.42 | 0.00 |
|  | 100739001 | *NXNL2* | nucleoredoxin-like 2 | 158046 | *NXNL2* | -7.45 | 0.00 |
|  | 100513220 | *GPR83* | G protein-coupled receptor 83 | 10888 | *GPR83* | -7.83 | 0.00 |
|  | 100157834 | *CDH17* | cadherin 17 | 1015 | *CDH17* | -8.34 | 0.00 |
